# Peatland Microbial Communities as Indicators of the Extreme Atmospheric Dust Deposition

**DOI:** 10.1007/s11270-015-2338-1

**Published:** 2015-03-18

**Authors:** B. Fiałkiewicz-Kozieł, B. Smieja-Król, T. M. Ostrovnaya, M. Frontasyeva, A. Siemińska, M. Lamentowicz

**Affiliations:** 1 Department of Biogeography and Palaeoecology, Faculty of Geographical and Geological Sciences, Adam Mickiewicz University, Dzięgielowa 27, 61-680 Poznań, Poland; 2 Faculty of Earth Sciences, University of Silesia, Będzińska 60, 41-200 Sosnowiec, Poland; 3 Department of Neutron Activation Analysis, Frank Laboratory of Neutron Physics Joint Institute for Nuclear Research, Dubna, Russian Federation; 4 Laboratory of Wetland Ecology and Management & Department of Biogeography and Palaeoecology, Adam Mickiewicz University in Poznań, Poznań, Poland; 5 Department of Meteorology, Poznan University of Life Sciences, Piątkowska 94, 60-649 Poznań, Poland

**Keywords:** Testate amoebae, Pollution, Fly ash particles, Proxy

## Abstract

We investigated a peat profile from the Izery Mountains, located within the so-called Black Triangle, the border area of Poland, Czech Republic, and Germany. This peatland suffered from an extreme atmospheric pollution during the last 50 years, which created an exceptional natural experiment to examine the impact of pollution on peatland microbes. Testate amoebae (TA), *Centropyxis aerophila* and *Phryganella acropodia*, were distinguished as a proxy of atmospheric pollution caused by extensive brown coal combustion. We recorded a decline of mixotrophic TA and development of agglutinated taxa as a response for the extreme concentration of Al (30 g kg^−1^) and Cu (96 mg kg^−1^) as well as the extreme amount of fly ash particles determined by scanning electron microscopy (SEM) analysis, which were used by TA for shell construction. Titanium (5.9 %), aluminum (4.7 %), and chromium (4.2 %) significantly explained the highest percentage of the variance in TA data. Elements such as Al, Ti, Cr, Ni, and Cu were highly correlated (*r* > 0.7, *p* < 0.01) with pseudostome position/body size ratio and pseudostome position. Changes in the community structure, functional diversity, and mechanisms of shell construction were recognized as the indicators of dust pollution. We strengthen the importance of the TA as the bioindicators of the recent atmospheric pollution.

**Electronic supplementary material:**

The online version of this article (doi:10.1007/s11270-015-2338-1) contains supplementary material, which is available to authorized users.

## Introduction

Ombrotrophic peatlands are an important archive of atmospheric pollution (Shotyk 1996). They give essential information about past and present changes of the environment, especially, when applying a multiproxy approach (Lamentowicz et al. 2013). Testate amoebae, unicellular protists, are one of the most abundant microbial group of the peatland ecosystem and represent soil food web structure as they are microbial top predators (Jassey et al. 2013a). These sensitive bioindicators are often used to reconstruct past hydrology and trophy (Swindles et al. 2014) as well as atmospheric pollution (Payne et al. 2012), because their shells are preserved in the peat after death. Recently, analysis of the TA functional traits revealed intriguing relationships between hydrology and pseudostome position (Lamentowicz et al. 2015); however, it has not been studied deeply how the atmospheric pollution affects the functional diversity of microbes. Furthermore, nothing is known about the response of these microbes to fly ash deposition resulting from coal combustion.

We investigated a peat profile from Jagnięcy Potok in the Izery Mountains, located within the so-called Black Triangle (Jędrysek et al. 2002; Szynkiewicz et al. 2008), the border area of Poland, Czech Republic, and Germany. This peatland suffered from an extreme atmospheric pollution during the last 50 years, which created an exceptional natural experiment to examine the impact of pollution on peatland microbes. We hypothesized that TA responded significantly to the dust deposition on the level of functional diversity, community structure, morphology, and physiology.

The objectives of our research are to (i) assess the level of pollution using geochemistry, (ii) determine the chemical composition of dust particles building the shell of TA using a SEM, and (iii) infer the response of testate amoebae communities and their functional traits to the toxic dust pollution.

## Methods

Seventeen dry samples of 1 g were used for epithermal neutron activation analysis (ENAA) to determine the concentrations of Al, As, Cr, Cu, Fe, Ni, Sr, and Ti. ENAA, which is effective in the simultaneous assessment of the content of important air pollutants (Frontasyeva and Steinnes 2005), was carried out in the IBR-2 pulsed fast reactor in Dubna (Russia). Samples of TA were prepared according to methods described by Booth et al. (2010). These samples were washed over sieves of 300 μm mesh size; the fraction below 300 μm was used for the analysis. The TA was counted at magnifications of ×200 and ×400 and identified using available literature (Grospietsch 1958; Mazei and Tsyganov 2006; Ogden and Hedley 1980). To determine the mineral composition of every peat sample and TA shells, SEM equipped with an energy dispersive system (Philips XL30 ESEM/EDS), allowing chemical analyses of individual peat components, was used (Smieja-Król and Fiałkiewicz-Kozieł 2014).

Hellinger-transformed testate amoebae community matrix was used as a response matrix (Legendre and Gallagher 2001). The redundancy analyses (RDA) to relate abiotic variables to biotic communities were employed. The significance of the model, axes, and variables was tested using a Monte Carlo test with 999 permutations. Computations were performed in R 3.0.1 (Team 2013) using the vegan package (Oksanen et al. 2011).

Three morphological traits were selected to infer the past pollution: metabolic status of the species, body size, and position of the shell aperture. The metabolic status of TA (presence/absence of endosymbiotic algae) indicates whether a specimen is mixotrophic (i.e., organisms able to combine autotrophic and heterotrophic nutrition) or heterotrophic. Metabolic status of TA was showed to cope with environmental settings such as moisture, temperature, and light (Jassey et al. 2011; Wilken et al. 2013). Those traits are expected to reflect community structure and disturbance connected with the pollution (Tsyganov et al. 2012; Lamentowicz et al. 2013). The position of the shell aperture (semi-continuous and variably coded as 0 = axial, 1 = terminal, 2 = sub-terminal aperture) revealed a gradient from exposed to protected aperture. We analysed the response of individual morphological traits in the peat-core by calculating the community-weighted mean (CWM) of each standardized trait (Lamentowicz et al. 2015).

## Results and Discussion

**Fig. 1:**
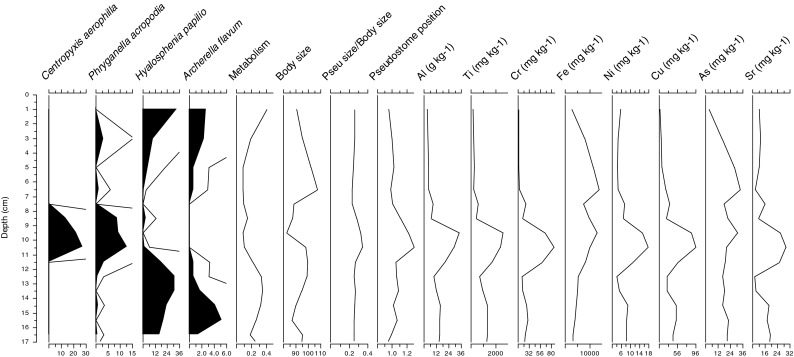
The most abundant testate amoebae, functional traits and elements concentration in the Izery along the peat profile

The strongest change in all determined variables was noticed at 10–12 cm peat layer (Fig. 1). A significant increase in the concentration of Al; 30 g kg^−1^ (by a factor of 10) and Ti; 2400 mg kg^−1^, followed by As; 20 mg kg^−1^, Cr; 85 mg kg^−1^, Cu; 96 mg kg^−1^ (factor of 5), Fe; 1 %, Ni; 18 mg kg^−1^, and Sr; 29 mg kg^−1^ was observed in comparison with values obtained in the lower part of the peat (29–30 cm). The concentrations were also distinctly higher than those in another Polish peatlands (Holynska et al. 1998; De Vleeschouwer et al. 2009). Anthropogenic particles, originating from brown coal combustion, dominate in the layer, constituting 66 % of all inorganic particles analyzed. These particles comprised irregular, spongy aluminosilicates (Al_2_O_3_ > SiO_2_) up to 50 μm large and glassy, often hollow spheroidal aluminosilicates (SiO_2_ > Al_2_O_3_) with diameters ranging between 0.8 and 10 μm (average 2.6 μm). The occurrence of both the spongy and glassy aluminosilicates and extreme concentration of titanium clearly indicate the impact of humans on investigated peatland (Smieja- Król et al. 2010; Smieja-Król and Fiałkiewicz-Kozieł 2014).

Mixotrophic (living in symbiosis with the green algae) (Gomaa et al. 2014) TA (*Archerella flavum* and *Hyalosphenia papilio*) species decreased, and then agglutinating (including extraneous mineral particles in shell) species—*Centropyxis aerophila* and *Phryganella acropodia—*became more abundant (Fig. 1). *P. acropodia* is usually regarded as an indicator of the low water table in *Sphagnum* peatland (Lamentowicz and Mitchell 2005) and *C. aerophila* is found in the mineral soil (Deflandre 1929). Considering functional traits of TA, we observed a shift from acrostomic to plagiostomic type of aperture that might suggest a transformation of the food web into the lower trophic level (Jassey et al. 2013b). Analysing the community-weighted means of specific functional traits, we found that pseudostome position is the most significant functional trait that determine the surviving of TA in the extreme pollution as well as increased input of the dust (Fig. 1). Also, the metabolism curve shows a decline of mixotrophic species that most possibly is connected with the light limitation. Furthermore, body size trait curve shows a decrease of large TA species during the pollution event. The considerable change within the functional diversity expresses the transformation of the food web triggered by the atmospheric pollution that shortened the food chain (Jassey et al. 2013a). This type of signal could have been interpreted as the dry shift (presence of dry TA indicators) according to community structure; however, possessing geochemical data we can be sure that it was generated by the air pollution.

Our analysis revealed a correlation between TA functional traits and measured elements (Supplementary Table 1). The highest number of high (*r* > 0.7, *p* < 0.01) Pearson correlation was obtained for pseudostome position/body size ratio and Al, Ti, Cr, Ni, and Cu. The second trait was pseudostome position that correlates highly with Al, Cr, and Ni. Metabolism revealed high but inverse correlation with Fe. Actually, such strong relationships support our inferences of the strong past impact of the atmospheric pollution on the soil microbes.

**Fig. 2:**
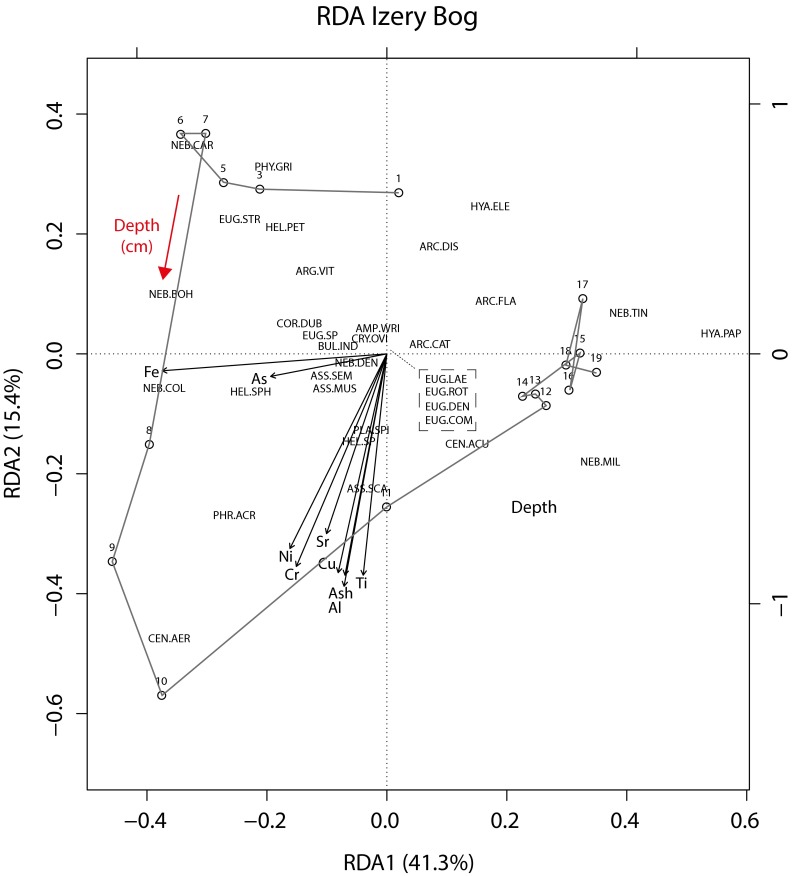
Redundancy analysis (RDA) of the pollution event in the Izery Mountains. Full model explains 75 % of the variation in species matrix. First axis explains 41.3 % and second 15.4 % of variance, both axes are significant (*p* < 0.001). The most significant variables in the model are Ti (*p* < 0.01) as well as Al and Cr (*p* < 0.01) (Supplementary Table 2). The *grey line* connects particular samples along the profile depth. Species names abbreviations: AMP FLA *Archerella flavum*, ARC CAT *Arcella catinus*, ARC DIS *Arcella discoides*, ARC FLA *Archerella flavum*, ARG VIT *Argynnia vitraea*, ASS MUS *Assulina muscorum*, ASS SCA *Assulina scandinavica*, ASS SEM *Assulina seminulum*, BUL IND *Bullinularia indica*, COR DUB *Corythion dubium*, CEN AER *Centropyxis aerophila*, CEN ACU *Centropyxis aculeata*, CRY OVI *Cryptodifflugia oviformis*, EUG COM *Euglypha compressa*, EUG DEN *Euglypha denticulata*, EUG ROT *Euglypha rotunda*, EUG LAE *Euglypha laevis*, EUG STR *Euglypha strigosa*, EUG SP *Euglypha sp.*, HEL PET *Heleopera petricola*, HEL SP *Heleopera* sp., HEL SPH *Heleopera sphagni*, HYA ELE *Hyalosphenia elegans*, HYA PAP *Hyalosphenia papilio*, NEB MIL *Nebela militaris*, NEB TIN *Nebela tincta*, NEB COL *Nebela collaris*, NEB DEN *Nebela dentistoma*, NEB CAR *Nebela carinata*, NEB BOH *Nebela bohemica*, PHR ACR *Phryganella acropodia*, PHY GRI *Physochila griseola*, PLA SPI *Placocista spinosa*

Redundancy analysis (RDA) revealed a significant (*p* < 0.001) response of TA to the extreme pollution event. Furthermore, it indicates the influence of ash content—Al, Cu, Cr, and Ti—at significant level (Fig. 2). Permutation test showed that titanium (5.9 %), aluminum (4.7 %), and chromium (4.2 %) are statistically significant and explain the highest percentage of the variance in TA data (Supplementary Table 2).

Copper and aluminum are potentially the most toxic elements; however, the form of their deposition is the most important factor that could strongly affect the microbial biodiversity. It is known that Al is especially mobile and toxic in acid pH (Kabata-Pendias and Pendias 1999). Although we were unable to achieve the chemical speciation of copper and aluminum, the large internal surface area of the spongy aluminosilicates and *Sphagnum* parts with absorbed aluminum indicate a possible release of this element to peatland briosphere and possible harmful effects.

**Fig. 3:**
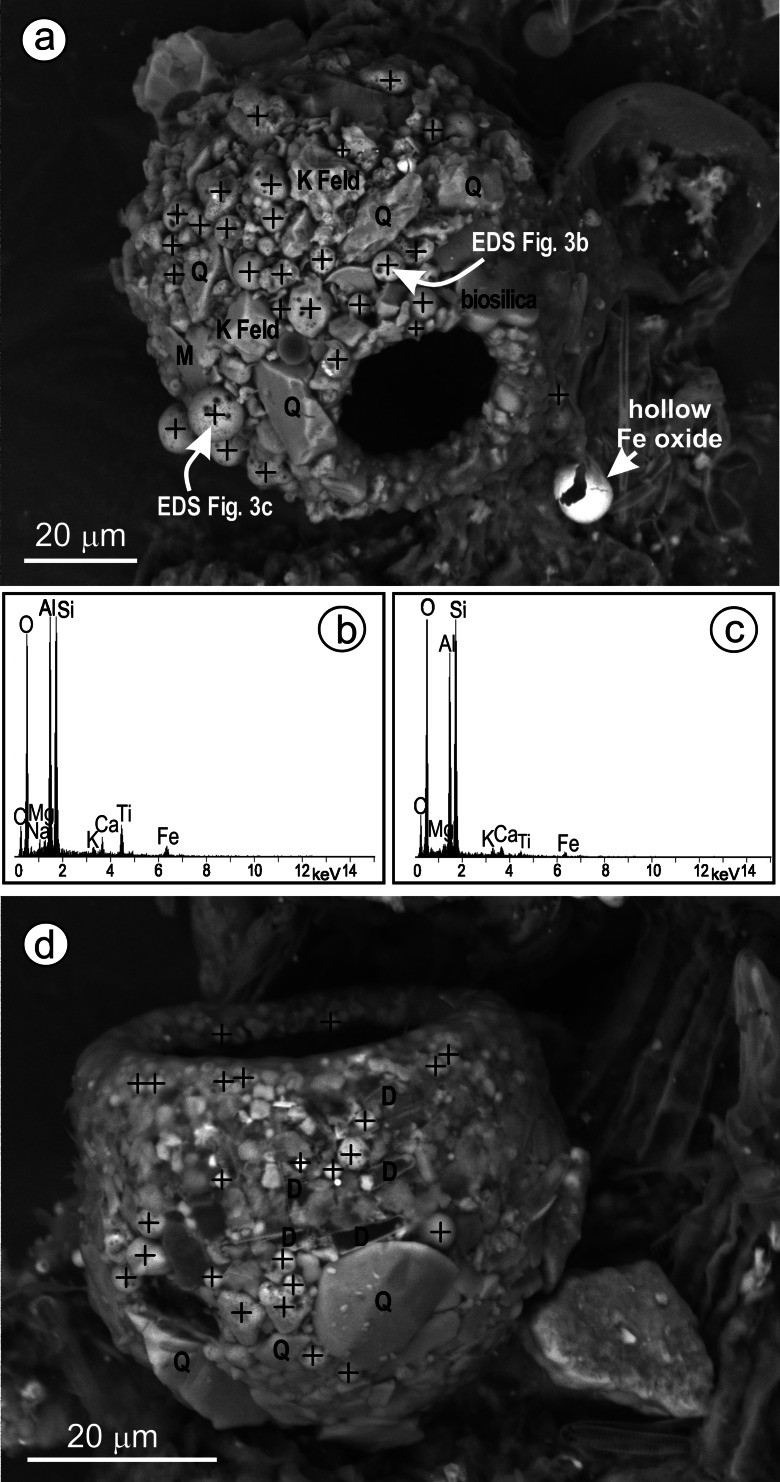
Scanning electron microscope images and EDS spectra of TA shells: **a** shell of *Difflugia sp.* covered by fly ashes; **b** and **c** EDS spectra of anthropogenic aluminosilicates from (**a**); **d** *Phryganella sp.*—anthropogenic particles that are built into the test. Anthropogenic particles are indicated by crosses, the identified natural particles are *Q* quartz, *F* feld–potassium feldspar, *M* muscovite, *D* diatom fragments. For more details about the SEM analysis of mineral composition of peat samples as well as testate amoeba shell please see Smieja-Król and Fiałkiewicz-Kozieł (2014)

*Centropyxis* and *Phryganella* used small spheroidal aluminosilicates for shell construction (Fig. 3). Silica-biomineralizing testate amoebae need Si in diluted form for shell construction (e.g., *Euglypha sp*.). As a consequence, they use potentially deleterious water, a fact which seems to be insignificant for life conditions of *Centropyxis* or *Phryganella*, but might be harmful for the construction of idiosomic and organic shells. Other paleo studies in Poland showed the development of *C. aerophila* during deforestation being a result of soil openness and erosion (Lamentowicz et al. 2009; Lamentowicz et al. 2007). However, in those studies the dust did not have toxic character.

The other possible explanation for limitation of some TA is the influence of ash on mixotrophic species by blocking the access to light as well as the supply of mineral particles (lacking in pristine *Sphagnum* peatland) for shell construction, which is the cause of changes in the composition of TA and preference of species using those particles.

Small species of *Difflugia* genus (not determined in standard light microscopy) were also recorded. They are covered by both types of anthropogenic aluminosilicates as well as rock-derived minerals (Fig. 3). However, further investigation is needed to assess if selection of minerals is species-linked.

## Conclusions

*C. aerophila* and *P. acropodia—*indicators of supply in mineral matter and atmospheric pollution in ombrotrophic peatlands, were distinguished. *Centropyxis*, *Difflugia*, and *Phryganella* possess different physiological mechanisms for shell construction using allochtonic particles. Al and Cu seem to be the most toxic elements and change the TA composition. Analysis of the concentration of toxic elements with mineralogical analysis and ecological traits of TA helps to better understand the response of microbial communities to environmental pollution. Finally, we strengthen the importance of the testate amoebae as the bioindicators of the recent atmospheric pollution.

## Electronic supplementary material

Below is the link to the electronic supplementary material.

Supplementary Table 1 Pearson correlation/significance matrix of particular elements and testate amoebae functional traits. Highly significant correlations exceeding 0.7 are presented in bold. Abbreviations: Met – Metabolism, BodSize – Body Size, PsdPos – Pseudostome Position, Psd/Body – Pseudostome Size – Body Size Ratio. (XLSX 13 kb)

Supplementary Table 2 Result of permutation test of each explanatory variable (number of permutations 999). Significance codes: 0 ‘***’ 0.001 ‘**’ 0.01 ‘*’ 0.05 ‘,’ (XLSX 10 kb)
